# Supplementary material for: Common molecular features of H3K27M DMGs and PFA ependymomas map to hindbrain developmental pathways
Source: Acta Neuropathol Commun. 2023 Feb 9;11:25. doi: 10.1186/s40478-023-01514-z (PMC9912509; doi:10.1186/s40478-023-01514-z)
Supplement: Supplementary file 1 — Additional file 1: Supplementary Figures 1–5. [file 40478_2023_1514_MOESM1_ESM.pdf]

Supplementary Information

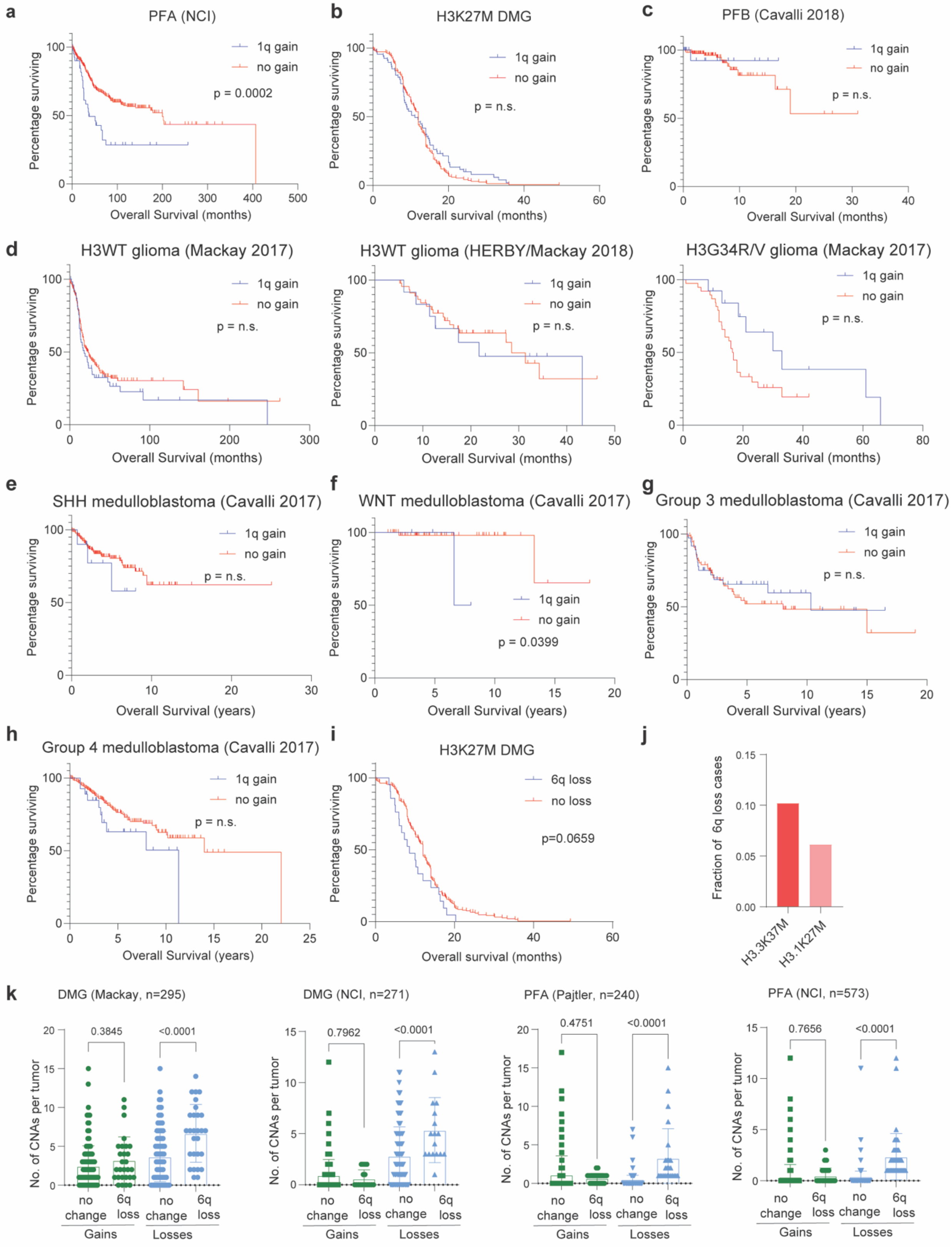

Supplementary Fig. 1: H3.1K27M and EZHIP-DMGs share key copy-number alterations with PFAs

a Overall survival (months, X-axis) analysis of PFA ependymomas from the NCI cohort with (n=41) or without (n=365) 1q gain.

b Overall survival (months, X-axis) analysis of H3K27M DMGs with (n=66) or without (n=178) 1q gain.

c Overall survival (months, X-axis) analysis of PFB ependymomas from Cavalli et al. (2018) with (n=20) or without (n=117) 1q gain.

d Overall survival (months, X-axis) analyses of 1q gain in other pediatric glioma cohorts from Mackay et al. (2017) or Mackay et al. (2018). Sample sizes with or without 1q gain are as follows: H3WT, Mackay 2018- n=54, 179; H3WT, HERBY- n=12, 46; H3G34R/V- n=13, 38.

e-h Overall survival (months, X-axis) analyses of 1q gain in other pediatric brain tumor cohorts (medulloblastoma subgroups) from Cavalli et al. (2017). Sample sizes with or without 1q gain are as follows: SHH (e)- n=10, 162; WNT (f)- n=4, 59; Group 3 (g)- n= 36, 77; Group 4 (h)- n=28, 236.

i Overall survival (months) analysis of H3K27M DMGs with (n=21) or without (n=216) 6q loss.

Data in S1a-i analyzed using Kaplan-Meier, Log-Rank test with 95% confidence intervals.

j Frequency of 6q loss (Y-axis) for subtypes of H3K27M DMG from published datasets depicted in 1a. H3.3 mutant (n=245) and H3.1 mutant (n=49).

k Number of copy number alterations (CNAs, Y-axis) in DMGs and PFAs. All four cohorts were segregated by tumors overall chromosomal gains (green) with (n=28-Mackay, 17-NCI:DMG, 25-Pajtler, 45-NCI:PFA) or without 6q loss (n=267-Mackay, 254-NCI:DMG, 215-Pajtler, 528-NCI:PFA), or overall chromosomal losses (blue). Data analyzed by two-sided, unpaired, two-tailed *t* test.

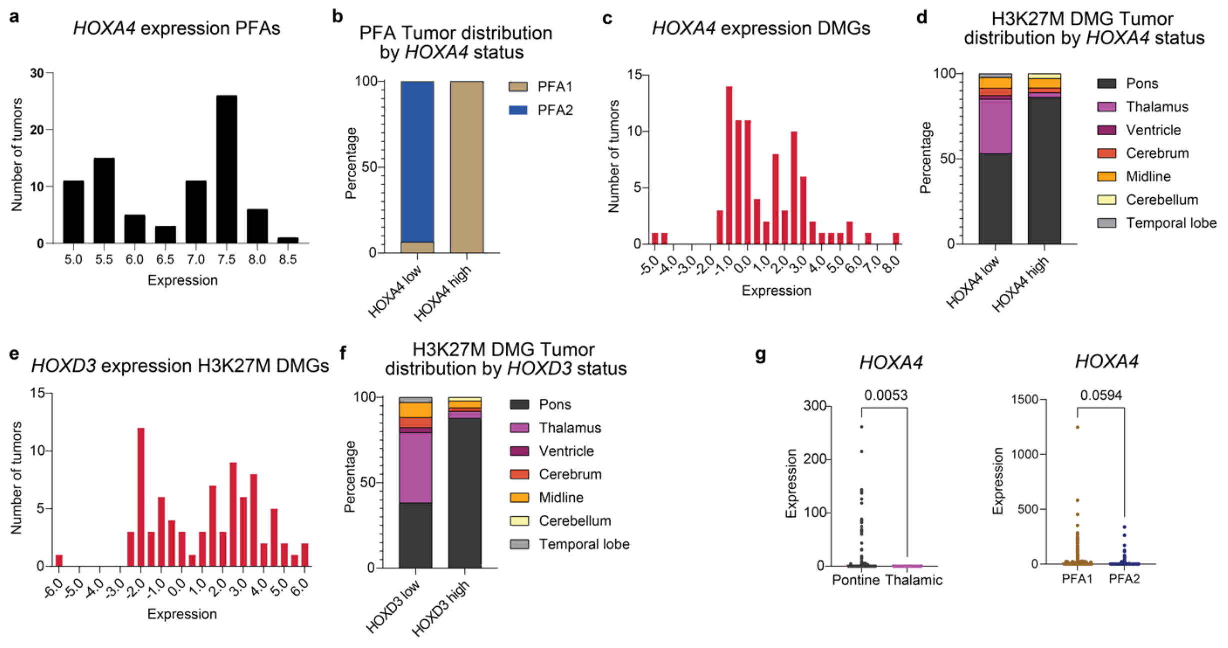

Supplementary Fig. 2: H3K27me3 commonly enriched genes in PFAs and H3K27M DMGs exhibit heterogeneity corresponding to tumor anatomic location

a Histogram of *HOXA4* expression values per tumor (X-axis = binned expression scores, Y-axis = number of tumors) in PFAs.

b Comparison of HOXA4-high (expression > 6 units) vs. -low (expression < 6 units) groups based on the local minimum between modes in DNA-methylation defined, anatomically distinct PFA1 (n=49) and PFA2 (n=29) subtypes of PFAs.

c Histogram of *HOXA4* expression values per tumor (X-axis = binned expression scores, Y-axis = number of tumors) in H3K27M DMGs.

d Comparison of HOXA4-high (expression > 1 unit) vs. -low (expression < 1 unit) groups based on the local minimum between modes with anatomic location of H3K27M DMGs (pons=65; thalamus=26; midline not otherwise specified (NOS)=5; cerebellum=1, and other =2).

e Histogram of *HOXD3* expression values per tumor (X-axis = number of values, Y-axis = bin center) in H3K27M DMGs. Note that *HOXD3* expression values were not available for PFAs.

f Comparison of HOXD3-high (expression > 0.5 units) vs. -low (expression < 0.5 units) groups based on the local minimum between modes with anatomic location of H3K27M DMGs (pons=65; thalamus=26; midline not otherwise specified (NOS)=5; cerebellum=1, and other=2). *HOXD3* expression values were not available for PFAs.

g Expression patterns of *HOXA4* in single-cell RNA-sequencing (scRNA-seq) experiments (Filbin et al. and Gojo *et al*.). Tumors were grouped by anatomic site (pons vs. thalamus for H3K27M DMG) or methylation-based subtype (PFA1 vs PFA2 for PFAs).

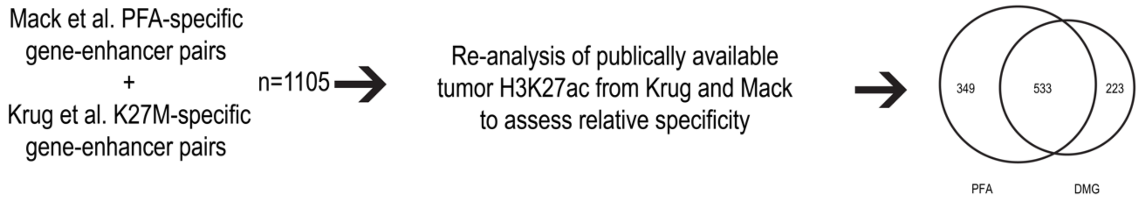

Supplementary Fig. 3: Enhancer signatures in PFAs and H3K27M DMGs show high expression in astrocyte-like tumor cells

Venn diagram depicting the construction of the shared and tumor-specific enhancer signatures.

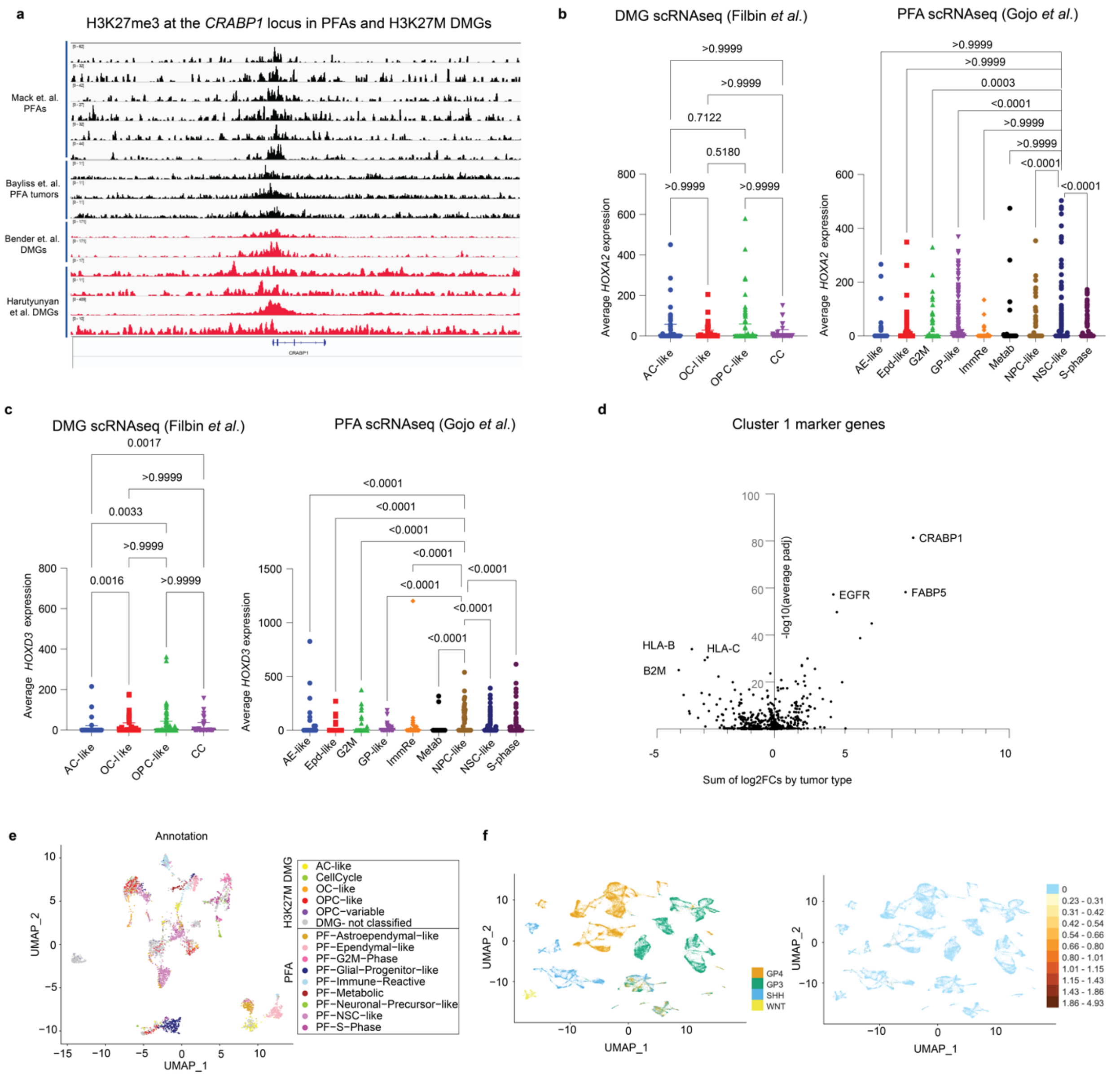

Supplementary Fig. 4. H3K27me3 enriched *CRABP1* is expressed highly in progenitor populations of both tumors

a H3K27me3 ChIP-seq tracks at the *CRABP1* locus for PFA (black, n=9, from Mack *et al*., and Bayliss *et al*.) and H3K27M DMG (red, n=6, from Bender *et al*. and Harutyunyan *et al*.) tumors.

b scRNA-seq expression of *HOXA2* grouped by cell-type in PFA and DMG datasets.

c scRNA-seq expression of *HOXD3* grouped by cell-type in PFA and DMG datasets.

d Volcano plot of Seurat conserved markers of Cluster 1 in the integrated H3K27M DMG/PFA scRNA-seq analysis from **Fig. 4g** (Y-axis = -log10-normalized average adjusted p-value across the two tumor sets, X-axis = sum of log2-transformed fold changes between Cluster 1 and all other clusters for the two tumor sets.

e Uniform Manifold Approximation and Projection (UMAP) embeddings of integrated scRNA-seq datasets of H3K27M DMG (Filbin) and PF ependymoma (Gojo) with cells colored by cell-type (AC-like, Astrocyte-like; OC-like, Oligodendrocyte-like; OPC-like, Oligodendrocyte precursor-like; CC- cell cycle; AE-like, Astroependymal-like; Epd-like, Ependymal-like; G2M, G to M cell cycle related; GP-like, Glialprecursor-like; ImmRe, Immune related; Metab, Metabolic; NPC-like, Neuronal precursor-like; NSC-like, Neuronal stem cell-like; S-phase, S-phase cell cycle.)

f Uniform Manifold Approximation and Projection (UMAP) embeddings of scRNA-seq data from all four subgroups of medulloblastoma (Riemondy et al.) with subgroup annotation (left) and *CRABP1* expression (right).

Kruskal-Wallis test followed by multiple comparisons analysis were used to analyze data in Figs S4b-c.

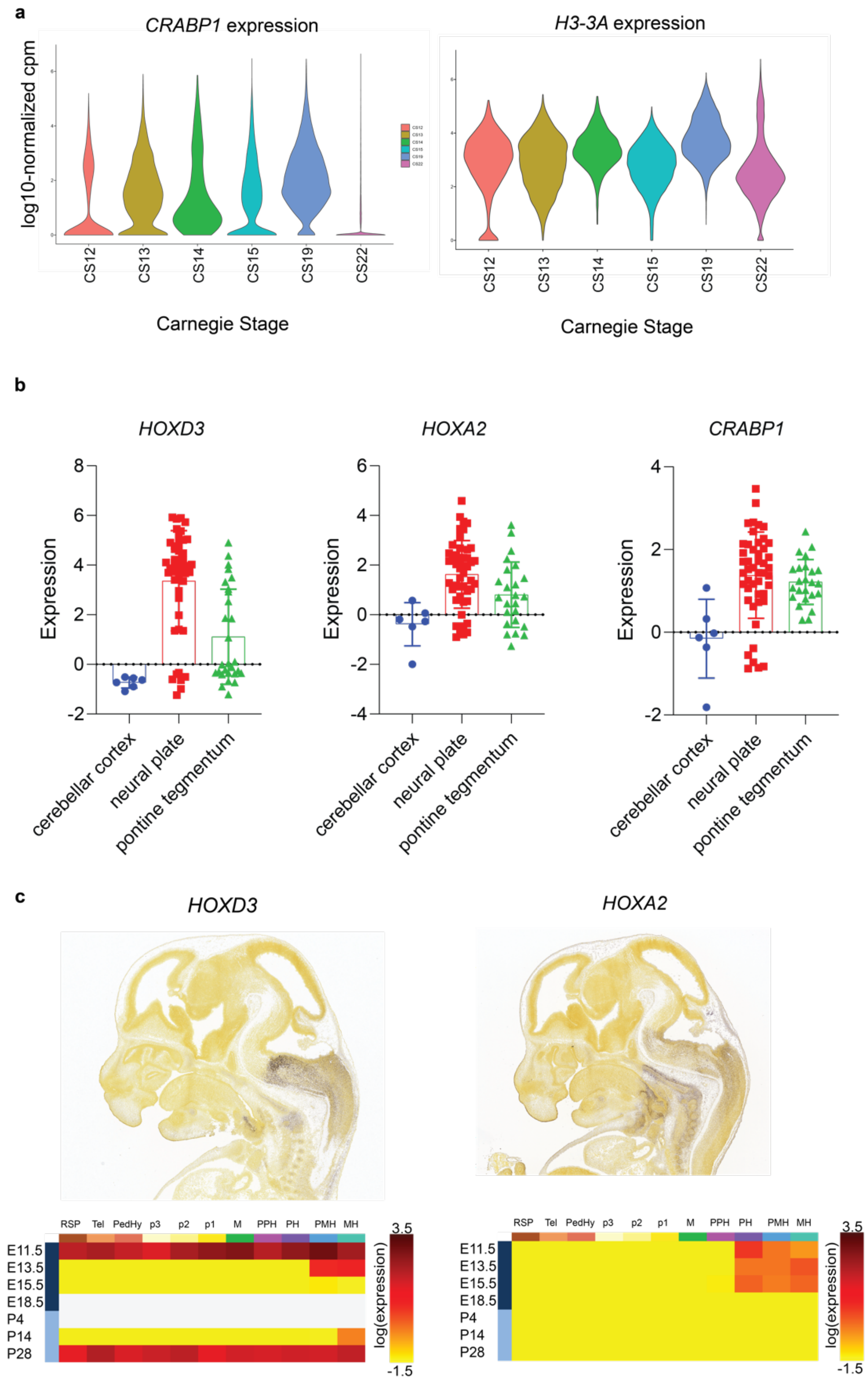

Supplementary Fig. 5. Common H3K27me3 signatures mirror human hindbrain brain developmental patterns

a First-trimester hindbrain *CRABP1* and *H3-3A* expression by Carnegie Stage (CS) of development

b Expression patterns of *CRABP1*, *HOXA2*, and *HOXD3* at 15 and 16 weeks post-conception in the BrainSpan prenatal LMD microarray dataset grouped by hindbrain substructures

c *HOXA2* and *HOXD3* expression patterns of the developing mouse from the Allen Brain Atlas. Top: *in situ* hybridization (ISH) staining for *HOXA2* and *HOXD3* in embryonic day 13.5 (E13.5). Bottom: Expression patterns of *HOXA2* and *HOXD3* across space and time in the developing mouse brain: RSP, rostral secondary prosencephalon; Tel, telencephalic vesicle; PedHy, peduncular hypothalamus; p3, prosomere 3; p2, prosomere 2; p1, prosomere 1; M, midbrain; PPH, prepontine hindbrain; PH, pontine hindbrain; PMH, pontomedullary hindbrain; MH, medullary hindbrain. Image credit: Allen Institute for Brain Science: https://developingmouse.brain-map.org/experiment/siv?id=100093342&imageId=101522680&initImage=ish, (*HOXD3* ISH) https://developingmouse.brain-map.org/gene/show/15209 (*HOXD3* heatmap), https://developingmouse.brain-map.org/experiment/siv?id=100030618&imageId=100662735&initImage=ish (*HOXA2* ISH) https://developingmouse.brain-map.org/gene/show/15174 (*HOXA2* heatmap).
